# Lay involvement in the analysis of qualitative data in health services research: a descriptive study

**DOI:** 10.1186/s40900-016-0041-z

**Published:** 2016-08-20

**Authors:** S. Garfield, S. Jheeta, F. Husson, A. Jacklin, A. Bischler, C. Norton, B. D. Franklin

**Affiliations:** 1 Centre for Medication Safety and Service Quality, Imperial College Healthcare NHS Trust, London, UK; 2 Research Department of Practice and Policy, UCL School of Pharmacy, Mezzanine Floor, BMA House, Tavistock Square, London, UK; 3 Pharmacy Department, Chelsea and Westminster Healthcare NHS Foundation Trust, London, UK; 4 King’s College London, Faculty of Nursing and Midwifery and Imperial College Healthcare NHS Trust, London, 57 Waterloo Road, London, SE1 8WA UK

**Keywords:** Patient and public involvement, Lay involvement, Qualitative data analysis, Open coding, Health services research

## Abstract

**Plain English summary:**

There is a consensus that patients and the public should be involved in research in a meaningful way. However, to date, lay people have been mostly involved in developing research ideas and commenting on patient information.

We previously published a paper describing our experience with lay partners conducting observations in a study of how patients in hospital are involved with their medicines. In a later part of the same study, lay partners were also involved in analysing interviews that a researcher had conducted with patients, carers and healthcare professionals about patient and carer involvement with medicines in hospital. We therefore wanted to build on our previous paper and report on our experiences with lay partners helping to conduct data analysis. We therefore interviewed the lay members and researchers involved in the analysis to find out their views.

Both lay members and researchers reported that lay partners added value to the study by bringing their own perspectives and identifying further areas for the researcher to look for in the interviews. In this way researchers and lay partners were able to work together to produce a richer analysis than would have been possible from either alone.

**Abstract:**

**Background** It is recognised that involving lay people in research in a meaningful rather than tokenistic way is both important and challenging. In this paper, we contribute to this debate by describing our experiences of lay involvement in data analysis.

**Methods** We conducted semi-structured interviews with the lay partners and researchers involved in qualitative data analysis in a wider study of inpatient involvement in medication safety. The interviews were transcribed verbatim and coded using open thematic analysis.

**Results** We interviewed three lay partners and the three researchers involved. These interviews demonstrated that the lay members added value to the analysis by bringing their own perspectives; these were systematically integrated into the analysis by the lead researcher to create a synergistic output. Some challenges arose, including difficulties in recruiting a diverse range of members of the public to carry out the role; however there were generally fewer challenges in data analysis than there had been with our previous experience of lay partners’ involvement in data collection.

**Conclusions** Lay members can add value to health services research by being involved in qualitative data analysis.

## Background

Recognition of the benefits of public involvement in health and social care research has been gathering momentum, and public involvement is increasingly identified as a prerequisite for funding [1]. Suggested benefits include improved quality of the results, more direct applicability of research to patients and improved translation into clinical practice [2]. Lay involvement has also been seen as ethically mandatory [2]. The UK Heath Research Authority and INVOLVE have issued guidance on the impact of public involvement on the ethical aspects of research and its potential contribution to make research more relevant, define what is acceptable to participants, improve the process of informed consent, improve the experience of participating in research, and improve the communication of findings to participants and the wider public [3].

However, there is also concern that current patient and public involvement is often tokenistic rather than placing a true value on patient input and involvement [1, 2]. Domecq et al. [2] carried out a systematic review of studies that described involving patients in the conduct, design and/or dissemination of research. They found many examples where lay people were involved in some aspects of research, such as agenda setting and protocol development, but that it was much less common for lay people to be involved in execution or translation of the research. It was concluded that research dedicated to identifying the best methods to achieve engagement was lacking [2].

We have previously reported our experiences of involving lay partners in data collection as an approach to engaging the public in health services research in a meaningful, less tokenistic way [4]. We identified both benefits and challenges to this form of lay involvement and considered how this contributed to the debate about the extent to which lay partners can carry out more extensive roles without losing their unique lay perspective [5, 6]. In a further phase of the same research project, lay partners were also involved in the analysis of transcripts of interviews with patients, carers, doctors, nurses and pharmacists. Previous papers have discussed the role of service users who were trained and employed as researchers in data analysis [7–9]. In this paper, we build on these and on our previous work [4] by describing our experiences of lay involvement in data analysis and consider how this further informs the debate about the benefits and challenges of extending lay involvement.

## Methods

### Setting

The context of this work was a wider study of how inpatients can be involved in medication safety while in hospital and the potential role of electronic prescribing systems as a barrier or facilitator to this (the Inpatient Medication, Patient Relationships and Electronic SystemS [‘IMPRESS’] Study [10]). The IMPRESS study had a patient and clinical engagement group that met quarterly and included five lay members. Potential sources of recruitment for lay members had included hospital volunteers, patient support groups and students. However, none of these were successful and members instead were recruited through personal contacts. As part of the IMPRESS study, researchers carried out interviews with patients, carers and health care professionals in two English National Health Service hospital organisations, to explore their attitudes to inpatient involvement in medication safety [10]. Organisation 1 had implemented electronic prescribing (EP) 7 years prior to the study and Organisation 2 implemented EP during the course of the study. One third of the interviews were carried out at Organisation 1, one third prior to the introduction of EP at Organisation 2 and one third following introduction of EP at Organisation 2.

Following completion of these interviews, four lay members of the patient and clinical engagement group expressed an interest in being involved in analysis of the interview data, which we then facilitated. The lay partners contributed to the analysis on a voluntary basis.

### Training of lay partners in data analysis

A one hour open coding training session was held in a café in which the principles and instructions for open coding were explained to the lay partners. This was followed up by a written set of instructions being circulated by email to the four lay partners participating in the analysis. Two of the lay partners were not able to be present at the open coding training session due to other commitments and received the information via email only. Neither the training session nor the follow-up email included practice analysis.

### Procedure for carrying out data analysis for the main IMPRESS study

The lead researcher coded all IMPRESS interviews using Vincent’s London Protocol [11] as the coding framework. This includes a classification of contributory factors influencing safety in clinical practice: patient factors, task factors, individual healthcare professional factors, team factors, environmental factors and organisational factors [11]. Following our lay partners’ request to be involved in data analysis, an open coding team independently analysed a purposive sample of the IMPRESS interviews from the lead researcher without an a priori framework, as the open coding team expressed a wish to be unconstrained by pre-existing frameworks.

The open coding team comprised two research team members (one research nurse and one research pharmacist) and four lay members. The lay members had backgrounds in journalism, physiotherapy, education, customer services/management. All had been a hospital inpatient, or had a close relative who had been a hospital inpatient, in the last five years. Due to there being a delay in the implementation of EP at Organisation 2, the post-EP interviews took place a year after the other interviews. There were therefore two open coding sessions: one for the analysis of interviews at Organisation 1 plus pre-EP interviews at Organisation 2, and a later one for the post-EP interviews at Organisation 2. At the first open coding session the two researchers discussed the themes alongside the lay partners, with the lay partners and researchers being considered equals in the discussion. At the second session, the only researcher present attended solely as an observer.

For each open coding session, a purposive sample of interview transcripts was emailed to each member of the open coding team, to include transcripts of interviews with each of the different stakeholders (patients, carers, pharmacists, nurses and doctors) and each of the organisations (Organisation 1, Organisation 2 pre-EP, Organisation 2 post-EP). Overall, 14 interviews of a total of 41 were sent to the open coding group. The open coding group then met together two months later to agree on a set of themes and sub themes. The lead researcher conducting the IMPRESS analysis was present at both open coding group meetings as an observer and was later sent the agreed themes, sub themes and example quotations by email. The lead researcher then integrated the themes and subthemes generated by the open coding group into the framework analysis. While the majority of themes identified by the open coding group fitted into Vincent’s London Protocol, an additional theme was added to the framework that was outside of Vincent’s London protocol. The lead researcher then emailed a document to the open coding group explaining how she integrated each theme they had identified, and invited further comment.

### Evaluation of lay role in data analysis

In December 2015 and January 2016, we conducted semi-structured interviews to explore the perspectives of the lay partners and the researchers who were involved in the analysis (the researchers being the lead researcher who analysed all the IMPRESS interviews plus the two researchers in the open coding group). Topics explored included previous experience of patient and public involvement in research, experiences of training for the open coding, experiences of carrying out analysis, and perceptions around benefits, challenges and recommendations for future studies.

#### Recruitment and data collection

The lead researcher emailed the four lay partners and the two other researchers who had been involved in the analysis to invite them to be interviewed at a mutually convenient time and location. The interviews were held face to face using a semi-structured interview guide. With consent of the respondents, the interviews were audio recorded. The lead researcher conducted the interviews with the lay partners and the other two researchers. Another member of the research team, who had not been involved in the open coding, interviewed the lead researcher.

#### Analysis of evaluation interviews

Interviews with lay partners and researchers were transcribed verbatim and coded using an inductive thematic approach [12]. Individual codes were generated and these codes were then combined into themes. QSR Nvivo 8 was used to aid in this process. Analysis was carried out by the lead researcher and reviewed by a second researcher in the team who had not been part of the open coding group. The second researcher checked both the themes identified and the coding. Any discrepancies were resolved via discussion.

## Results

All three researchers and three of the four lay partners were interviewed; the fourth lay partner, who was one of the two not present at the open coding training session, declined to take part without giving a reason.

### Previous experience

The lay partners and researchers had a range of previous experience in research and analysis. Two of the three lay partners involved in the open coding had previous experience of involvement in research projects, whereas the third had not. Two of the lay partners had experience of some form of data analysis as part of jobs they had held previously but the third had not been involved in any analysis before. All three researchers had experience of lay involvement in research projects but only one had previous experience in involving lay partners in data analysis and she indicated that this was still a somewhat ‘experimental’ rather than ‘established’ approach.

The way I like to work as a researcher. I always try and involve patients…. So I’ve worked a lot with people, both developing research proposals, but also then with getting them to comment on projects as they go along, writing patient-facing materials, or at least checking them if they’re not actually writing them. And then increasingly experimenting with getting lay involvement in both data collection and data analysis. (Researcher 2)

### Training

Lay partners and researchers expressed overall positive views on the training provided and were of the opinion that the informal setting of a café had worked well and that the training itself had been very interactive. The written instructions sent round after the meeting was also reported to be helpful and straightforward and sufficient for those unable to be present at the training session.

The view of the all the lay partners and researchers involved with open coding was that the training had been less detailed than the trainees had anticipated. The dominant view was that this reflected the fact that open coding was a simple and straightforward process, and that not a lot of training was required or would be helpful to achieve the objective of bringing out unique lay perspectives.

‘I think they almost wanted a template or a sort of proforma of this is how you do it. And I think as we suggested, it really is a matter of ‘read it through and write down what comes to mind’, and they sort of almost thought, ‘oh is that all? But I think that’s appropriate actually because I think if you do constrain people’s thinking you get what you ask for. Whereas if you allow them to be completely open then you’re preparing yourself to be surprised, which is great.’ (Researcher 2)

‘The open coding training was good, it was quite sort of sparse on the day and it was also quite, it seemed to me to be quite casual, but I think that's just the flexible nature of open coding.’ (Lay person 2)

However, one of the researchers expressed a concern that the scientific and systematic nature of qualitative analysis had not been communicated adequately, and another was of the view that it would have been helpful for the open coding group to have known more about how the open coding related to the framework analysis carried out by the lead researcher.

‘I kind of felt it was being presented in a less scientific way to the way I would do coding. … There was this talk about it is all very subjective, everyone will do this differently, whereas for me, when I am doing coding, there are all these reliability processes, which this was a part of. …. I think I would have wanted them to understand more the context. I think the fact what they were doing, like open coding, was really good, and the best way to do it, and I think it didn’t matter that we didn’t do it all so systematically, because [researcher] had done that and they were contributing their perspective to the theme. But I think it would have been helpful if they could understand a little bit more about how their analysis and [researcher’s] analysis would begin to gel together, and where the differences might be, and why the two were really important.’ (Researcher 3)

‘So, if I were starting again I would have … got some people to, offered the opportunity for them to code against [researcher’s] framework, because I think they could have really understood framework more. If you were going to add even more value it would be that, because then you'd get a cohort of people that really got the framework in the way they got the open coding and everything would be positive about that.’ (Researcher 1)

One of the lay partners was also of the view that a hands-on trial run of open coding may have been helpful and one of the researchers said that they had done that in lay open coding training sessions she had run previously.

### Benefits

All the lay partners reported an overall positive experience of carrying out the open analysis and that it had been an enjoyable experience.

‘I thought it was going to be a little bit of a chore … but actually I really enjoyed the analysis work.’ (Lay person 3)

In addition, one was of the view it had provided them with transferable skills for other research projects.

‘That was very good, first of all because I didn’t know about open coding at all, so this was a discovery. And the training was done extremely well by a member of the research team, and the whole process was really very enjoyable and very easy to do. And in fact one can wonder why it’s not done more often in [health] service research. Also because in fact open coding is actually quite simple and once one has done it once for a specific study then it becomes even easier for the next piece of work.’ (Lay person 1)

Both lay partners and researchers were of the view that lay involvement in the analysis had increased lay engagement with the project as a whole.

‘Well, I felt I enjoyed that and I felt very involved in the project and very involved with the other people that were doing similar things to me as well as the researchers, because you know, researchers had done the interviews. So, it increased my feeling of belonging if you see what I mean.’ (Lay person 2)

The researchers expressed the view that lay involvement in open coding had contributed greatly to the project and analysis by adding new perspectives to the findings and an enhanced understanding of the data as seen in the following example:

I think it definitely enriched our understanding of what was in that data… It kept us as researchers having a more open mind, being open to more possibilities. I think they did add a new perspective in terms of what they saw or the emphasis on what they saw from what maybe we had seen. So I think it was definitely additive and added a different dimension to some at least of the findings.’ (researcher 2)

It became apparent that lay and researcher contributions to the analysis had worked synergistically. For example the two researchers and lay partners in the first open coding session identified the theme of ‘contradictions’ where healthcare professionals said that they supported involvement of patients but then later expressed negative views about patient involvement. The lead researcher then did some extra analysis of the whole data set and found that these apparent contradictions reflected how healthcare professionals do support some involvement but that the extent of this is limited.

‘What was very important is that everyone’s contribution could be inserted in a very natural way; it wasn’t someone providing a contribution in an isolated – like in a tunnel, which sometimes can happen. It was basically a very rich mosaic of contribution and views all meshing together to provide a very, very rich outcome.’ (Lay person 1)

‘I did do a bit of extra analysis as a result of it, so I took their theme and made sure I had analysed it systematically for the whole data.’ (Researcher 3)

### Challenges

Researchers and lay partners expressed the view that lay involvement in analysis meant an additional time commitment from both lay partners and researchers in terms of the training, carrying out the analysis and then meeting together to discuss it. This was not necessarily thought of as something negative but as something that needed to be planned in.

‘I quite liked the fact that it was time-consuming and it made you concentrate for an hour on … [each] separate one as it were. So, I wouldn't want to alter the time that I put in, but what was useful to me as well was knowing when the stuff was going to come into my inbox, it was knowing that you know, they were expected on Tuesday next week sort of thing so that I could plan some time in the coming weeks in order to be able to do it at a quiet time when nothing else was going on in the house.’ (Lay person 2)

Another challenge, raised by the researcher who also had previous experience of lay analysis, was that lay partners may bring their own experiences rather than simply analysing what is in the data. While it was acknowledged that the purpose of lay involvement was to bring in new lay perspectives, the line may be crossed when perspectives from outside the data are brought in rather than using experiences to help interpret the data itself.

‘It’s very legitimate to interpret on the basis of personal experience, we all do that … I really don’t believe you want to sort of bracket your previous experience and I don’t think it’s desirable, I think usually. But I think … it’s a very fuzzy line actually between what enriches your analysis and what takes you completely off-message and off what’s actually in the data.’ (Researcher 2)

Yet another challenge identified was that of recruiting a diverse group of lay partners to conduct lay analysis who would be representative of the general population.

‘The main difficulty was actually the fact that we weren't able to recruit a broader range of people. But, I don't know that we could've done anything else to change that; we did everything we possibly could, I don't think we left a stone unturned.’ (Researcher 1)

### Suggestions for future lay involvement

While lay partners and researchers were all in favour of future lay involvement in analysis, there were also opinions expressed that this would not be suitable for all projects. One researcher was of the view that this would be more suitable for qualitative rather than quantitative analysis, another suggested that it may not be suitable in some clinical areas such as palliative care or where data may be sensitive, confidential or distressing for lay partners. A lay partner thought it would be more suitable for health services research than for clinical research.

A range of views were also expressed as to who the lay partners involved in analysis should be. One of the researchers expressed the opinion that that if lay partners were continually involved in research projects, they may start ‘coming to the data with a researcher’s hat on.’ All the lay partners who were involved in the interview analysis had also been involved in the earlier observations conducted as part of the IMPRESS study. The lay partners thought there was a natural progression from the observations to the interview analysis and that the same themes emerged.

‘Well, sometimes I felt as though I could identify the patient and sometimes I felt as though I could identify the doctors and nurses and it was very similar to the observations that we did and it reflects the experience quite well I thought. So, in a sense there were no surprises in the interviews, because they followed the format that I had seen, I'd observed on the ward.’ (Lay person 2)

One of the researchers was of the opinion that less training had been needed because the lay partners already knew about the project. However, one of the lay partners expressed the opinion that although following on from the observations made the analysis easier, it would have been interesting to have had some lay partners conducting analysis who had not been involved in the observations.

‘One of the things I guess that you’re going to have – and I was aware as I was doing it, is you’re going to have your own biases from the observation work and you’re almost looking for those to come out in the analysis work, whereas if you came at it not having done the observations you might spot some different trends or some different issues. So I think it would probably be quite interesting to do a combination of people that have been involved in the observations and people that haven’t and see if there’s any fundamental difference.’ (Lay person 3)

Another question that arose in one of the researcher interviews was whether in the future it would be better to have a mixture of healthcare professionals and lay partners in the open coding group, as in the first open coding session, or to have lay partners only, as in the second session. The lead researcher was of the view that either would be of value and that it would depend on the desired outcome. She reported that she was able to integrate the findings into the coding structure more easily from the first but that she was able to incorporate lay perspectives into the final publication in both cases.

‘I think in terms of the output I found the first one easier to integrate more quickly with my analysis, so if you wanted the analysis to be totally integrated in, the first one would work better. I think if you just wanted to incorporate the lay analysis, but maybe you wouldn’t be able to do it in quite a systematic way as I have described … then you could use the second one. So I think you would need to think about what your overall objectives were, what was more important to you, to have it completely lay? Or to have it a bit more structured?.’ (researcher 3).

A fourth suggestion for the future was to train lay partners on how to do qualitative analysis electronically rather than on paper to avoid large amounts of printing.

## Discussion

This study suggests that involving lay partners in qualitative data analysis can have similar benefits to those identified when involving them in data collection [6] in terms of lay motivation and engagement and added value to the research. Researchers were of the specific view that involving lay partners in the analysis had enhanced the knowledge gained through the study. The study therefore suggests that involvement in analysis is another way of engaging lay partners in research and overcoming the challenge of tokenistic involvement. However in this study, lay involvement in analysis was in addition to lay involvement in data collection. Therefore it is not known whether involvement in analysis alone, without previous involvement in data collection, would have the same benefits. Although one of the interviewees was of the view that lay involvement may not be suitable for some areas such as palliative care, carers have been successfully involved in a previous palliative care study, albeit in data collection rather than analysis [13]. Within this study, patient and public involvement in analysis potentially made the research more relevant to the public, in line with ethical guidelines [3], by adding new perspectives to the data.

There were fewer challenges identified in involving lay partners in analysis than had been experienced in data collection [4]. The lay partners did not have direct patient contact and received only anonymised interview transcripts. This meant that the concerns relating to information and research governance that arose in data collection did not arise in analysis. While lay involvement in analysis was time consuming for lay partners and some extra researcher time was required, this did not appear to be as big a challenge as for involvement in data collection. The lay partners were perhaps less worried about the time taken up as it was all productive time, in comparison to the time needed to carry out required paperwork and time spent waiting on the wards to observe healthcare professionals. The researcher resource required was also less as researchers did not need to be present while the lay partners did their own individual analysis, whereas they had needed to be present on the ward while lay partners conducted every observation [4]. There were still some challenges remaining such as lack of diversity among lay partners, as discussed previously [4].

While based on a small sample, this study helps further inform the debate as to how much training to give lay partners in order to ensure that they can carry out more extensive research tasks while keeping their unique lay perspective. In previous studies service users have been trained and employed as researchers and received extensive training [7-9] and were thereby able to add value to the research. In this study, lay partners received minimum training on open coding and came to the analysis in a very open way. This worked well as the researcher leading on the analysis was then able to add the lay themes identified to the analysis in a systematic way. Although this could be done in a more structured way when researchers were also involved in the open coding, it was also possible to integrate lay themes into the final publication even where researchers were not directly involved in the open coding group. While the lay partners made a strong contribution to the analysis, both researchers and lay partners identified that the final output was a synergy of researcher and lay perspectives where individual contributions could no longer be easily separated. As with data collection, lay partners were able to work in true partnership with researchers without losing their own perspective. This is a way of overcoming the challenge that Martin and Finn [5] and Ives [6] have identified in allowing lay partners to contribute to research in a more extensive and meaningful way without losing their own ‘outsider’ view. However, in the present study all the lay partners who were involved in analysis had also been involved in observations and were already integral partners in the research. One of the lay partners reflected that they therefore may not have been true outsiders as they approached the analysis. The findings also suggest that while the approach worked and the lay partners identified their perspectives in the resulting publication, it may have been helpful for the lay partners to have understood more about the process of how their open coding was integrated into the final analysis.

This study also suggests that lay partners may benefit from access to qualitative software packages such as Nvivo. In this study we did not have sufficient licensing for Nvivo to allow the lay partners to each have access but this should be considered in further funding applications that include lay involvement in analysis.

### Limitations

This study was limited to interviews with the small number of lay partners and researchers who were involved in one research project. We only had a pool of seven people to invite to interview, six of whom agreed to participate. It is there unclear how generalisable the findings are. However, only having a small number of interviews allowed us to conduct an in-depth analysis. The researchers conducting the interviews were part of the research team rather than external to the study which meant that there was potential for social desirability bias. However, given the scarcity of studies addressing this issue, these findings can help inform future patient and public involvement in studies of this type. Future evaluations of this kind, with independent researchers, will be able to test cumulative validity for the study findings. Future research should evaluate involving lay partners in data analysis without their prior involvement in data collection.

## Conclusion

This study suggests that involving lay partners in the open coding of qualitative data can increase lay engagement and motivation and may add value to health services research. By involving both lay partners and researchers in analysis it is possible to allow lay partners to bring their own perspectives to the data and ensure the analysis is rigorous and robust.
